# M6A-related lncRNAs predict clinical outcome and regulate the tumor immune microenvironment in hepatocellular carcinoma

**DOI:** 10.1186/s12885-022-09925-2

**Published:** 2022-08-09

**Authors:** Bo Chen, Zhan Yang, Zhichao Lang, Qiqi Tao, Rongrong Zhang, Yating Zhan, Xuantong Xu, Kai Zhu, Jianjian Zheng, Zhengping Yu, Suhui Yu

**Affiliations:** 1 Key Laboratory of Diagnosis and Treatment of Severe Hepato-Pancreatic Diseases of Zhejiang Province, The First Affiliated Hospital of Wenzhou Medical University, Wenzhou, 325000 China; 2 Department of Hepatobiliary Surgery, The First Affiliated Hospital of Wenzhou Medical University, No.2 fuxue lane, Wenzhou, 325000 Zhejiang People’s Republic of China

**Keywords:** Hepatocellular carcinoma, N6-methylandenosine, Long noncoding RNA, Prognosis, Tumor microenvironment, ceRNA network

## Abstract

LncRNA N6-methylandenosine (m6A) modification has been shown to be associated with the constitution of the tumor microenvironment (TME) and tumorigenesis. It’s essential to understand the mechanisms of lncRNA m6A modification in hepatocellular carcinoma (HCC) and identify relative prognostic predictors to guide therapy and explore potential therapeutic targets. Pearson correlation analysis was performed to identify m6A-related lncRNAs in 374 patients with HCC. Unsupervised cluster analysis of the potential m6A-related lncRNA-based HCC subtypes was conducted, followed by the concurrent analysis of their relationship with TME characteristics, immune checkpoints, immune features, and prognosis through single sample gene set enrichment analysis and ESTIMATE algorithm. Cox regression analyses were performed to screen prognostic m6A-related lncRNA, construct an m6A-related lncRNA signature (m6A-RLRS), and establish an integrated nomogram for the prognosis of patients with HCC. We identified 61 m6A-related lncRNAs and two HCC subtypes defined by consensus cluster of m6A-related lncRNAs with distinct clinical features. Progression-free survival (PFS), three TME-related scores, 15 immune-associated gene sets, and two immune checkpoints expression were found to be significantly different among the two subtypes. Twenty-five prognostic m6A-related lncRNAs were determined, four of which were included to establish an m6A-RLRS with favorable discrimination, and the signature was validated in the validation set and an independent FAHWMU cohort (*n* = 60). Furthermore, a novel nomogram combining signature and clinical predictors was generated with a C-index of 0.703, and an original ceRNA regulatory network consisting of 9 lncRNAs, 28 miRNAs, and 75 target mRNAs was constructed. Finally, the differential expression of four m6A-related lncRNA was verified by qRT-PCR. In conclusion, m6A-related lncRNA prognostic signature and molecular subtype contributes to accurately predict the prognosis of HCC and provide potential novel therapeutic targets.

**Supplementary Information:**

The online version contains supplementary material available at 10.1186/s12885-022-09925-2.

## Introduction

Hepatocellular carcinoma (HCC), accounting for about 90% of all primary liver malignancies, remains the sixth most common cancer and the third primary cause of cancer-associated death (about 906,000 new events and 830,000 deaths) in 2020 [1, 2]. Surgical excision, liver transplantation, and ablation are the effective curative strategies for patients with early -stage disease; however, 14–36.7% of HCC patients present with extrahepatic metastasis at initial clinic diagnosis [3, 4]. Recently, new therapeutic strategies, including targeted agents and immune checkpoints inhibitors, have been found to improve the prognosis of patients with advanced HCC [5, 6]. Nevertheless, most patients are still prone to poor prognosis due to recurrence, serious side effects, and chemoresistance [7], with a five-year survival rate of 12–18% [8, 9]. The advent of next-generation sequencing technologies has uncovered vital tumor-inducing genes and associated oncogenic pathways in HCC [10], and genetic signatures, taking into account the tumor microenvironment (TME) and heterogeneity, to predict treatment response and prognosis [11]. Hence, there is an urgent need to investigate the underlying molecular mechanisms and novel biomarkers for patients with HCC, which can be utilized for the accurate diagnosis and to improve prognosis.

N6-methylandenosine (m6A) is the most common methylation modification in RNA that significantly impacts RNA metabolism, which plays a crucial role in RNA splicing, nuclear output, and translation decay [12–14]. Several studies have shown that m6A modification acts by regulating gene expression, inflammatory response, cell differentiation, and carcinogenesis [15–17], making it a novel biomarker for the diagnosis, prognosis, and therapy of patients with a malignant tumor. Chen et al. showed that METTL3 promotes the proliferation and migration of HCC cells via the YTHDF-2 related pathway, and its knockdown inhibits cancer progression [18]. Li et al. showed that HIF-1alpha-induced YTHDF1 drives autophagy and progression by facilitating ATG2A and ATG14 translation in an m6A -dependent manner [19]. WTAP has been identified to be significantly upregulated in HCC and promotes malignancy of HCC through m6A-based epigenetic silencing of ETS1. These findings indicate that m6A modification of mRNA is involved in HCC carcinogenesis with significant prognostic values [20].

In recent years, numerous studies have pointed out that m6A modification can affect the splicing and maturation of noncoding RNAs (ncRNA). Also, long noncoding RNA (lncRNA), a subgroup of noncoding RNAs > 200 nucleotides in length, can be regulated by m6A methylation involved in cancer [21]. The m6A modification is known to play a dominant role in promoting lncRNA X-inactive specific transcript (XIST)-mediated transcriptional silencing. Zhang et al. revealed that ALKBH5 promotes metastasis and invasion of gastric cancer by reducing methylation of LncRNA NEAT1 [22]. LncRNA DANCR has been reported to serve as a novel target for IGF2BP2 through m6A modification and facilitates the pathogenesis of pancreatic cancer [23]. LINC00958 has been found to be upregulated by m6A methylase METTL3 and promotes HCC invasion and migration by interacting with miR-3619-5p [24]. However, the specific function of m6A modification in lncRNA regulation in HCC as well as lncRNA-dependent mechanisms to affect the occurrence and development of HCC is still unclear.

Here, we integrated the genomic information of 374 HCC tissues to comprehensively evaluate the m6A modification patterns of lncRNA and correlated with TME characteristics and immune features. The role of m6A-related lncRNA was determined by survival, differential, correlation analyses. we identified two HCC subtypes defined by consensus cluster of m6A-related lncRNAs with different prognostic outcomes and clinicopathological features. Moreover, we constructed and validated an m6A-related lncRNA signature (m6A-RLRS) and a nomogram combining the signature and clinical variables to assess the prognosis of patients with HCC. Finally, a competing endogenous RNA (ceRNA) network comprising of 9 m6A-related lncRNAs, 28 miRNAs, and 75 targeted mRNAs and enrichment analysis were implemented to detect the potential molecular mechanisms.

## Materials and methods

### Data collection

RNA-sequencing (RNA-seq) expression profiles of the HCC cohort were obtained from the TCGA database (https://portal.gdc.cancer.gov/repository), and the corresponding clinical information was acquired from the cBioPortal database. We normalized the RNA-seq data (FPKM values) to log2 (FPKM + 1), resulting in the identification of 374 HCC and 50 normal tissues for genomic expression analysis. Moreover, after excluding the patients with overall survival (OS) < 30 days, 343 patients were obtained, which were then randomly divided into a training set (70%) and a testing set (30%) for the further survival-related analysis. Two hundred forty-three patients in the training set were used to construct the models, while the remaining 100 patients in the testing set were used for validation. Table 1 shows the characteristics of patients in the training and testing sets. Meanwhile, a local serum set (FAHWMU set, *n* = 60) from the First Affiliated Hospital of Wenzhou Medical University (FAHWMU) was utilized to further verify the accuracy of risk models. The distribution of clinical confounding factors was not significant different between the FAHWMU set, training set and validation set (Supplementary Table 1). The lncRNA-to-miRNA regulatory relationships were downloaded from the miRcode database, and miRNA-to-mRNA regulatory relationships were acquired from miRDB, miRTarBase, and TargetScan databases.

**Table 1:** Baseline clinical characteristics of HCC patients

	Training group	Validation group	χ2/t	P
Age, years	59.4 ± 13.0	59.4 ± 13.59	0.017	0.986
Sex			0.097	0.755
Male	167	67		
Female	76	33		
Tumor Grade			4.302	0.352
I	32	21		
II	117	44		
III	83	29		
IV	8	4		
Unknow	3	2		
T stage			0.600	0.985
I	121	48		
II	58	26		
III	53	21		
IV	9	4		
Unknow	2	1		
N stage			1.214	0.546
N0	172	68		
N1	3	0		
Unknow	68	32		
M stage			3.121	0.176
M0	180	65		
M1	2	1		
Unknow	61	34		
TNM stage			8.088	0.076
I	117	45		
II	56	21		
III	59	21		
IV	2	1		
Unknow	9	12		

### Identification of m6A-related lncRNAs

A total of 21 m6A regulators were identified according to the publications, including nine methyltransferases (WTAP, RBM15, RBM15B, ZC3H13, KIA1499, METTL16, METTL14, and METTL3), two demethylases (ALKBH5 and FTO), and eleven m6A-binding proteins (YTHDC1-2, YTHDF1-3, IGF2BP1-3, HNRNPA2B1, HNRNPC, and RBMX). Pearson correlation analysis was performed among 374 patients with HCC to identify m6A-related lncRNAs with the “limma” package, and lncRNAs with R > 0.5 and *P* < 0.001 were selected for further study.

### Survival, differential, and correlation analyses of m6A-related lncRNAs

Since the expression data and OS time of 343 patients were integrated, the univariate Cox regression analysis was performed to identify OS-related m6A-related lncRNAs. Then, the differential expression of prognostic m6A-related lncRNAs were evaluated by the Wilcoxon signed-rank test with the “limma” package in 374 HCC and 50 normal tissues. Moreover, the specific m6A regulator-to-lncRNA correlations were generated using pearson correlation analysis. A *P*-value of 0.05 was considered to be of statistical significance in these tests.

### Unsupervised cluster analysis to identify HCC subtypes based on m6A-related lncRNAs

The unsupervised cluster analysis was used to classify 343 HCC cases based on the 61 m6A-related lncRNAs using the “ConsensusClusterPlus” package to explore the underlying distinct m6A modification patterns of lncRNAs. The number of HCC subtypes and stability were determined by the Gap statistic and Elbow method. Then, the associations between HCC subtypes and prognosis, including progression-free survival (PFS) and OS, were evaluated by the Kaplan–Meier (K-M) survival curves. Additionally, the immune features and TME characteristics among the distant m6A-related lncRNA-based subtypes were compared using the Wilcoxon signed-rank test, including 3 types of TME-related scores and 29 immune-related gene datasets, which covered the aspects of enrichment levels of immune infiltration cells and pathways in the tumor samples. Additionally, three TME-related scores (Immune score, Stromal score, and Estimate score) were calculated by the “ESTIMATE” package, and 29 immune-associated gene sets were generated by single-sample Gene Set Enrichment Analysis (ssGSEA) with “GSEABase” package.

### Establishment and validation of the m6A-related lncRNA signature (m6A-RLRS)

The prognostic m6A-related lncRNAs identified above were brought into the multivariate Cox regression analysis to determine the vital lncRNAs to comprehensively understand the clinical significance of m6A-related lncRNAs in HCC. Based on the critical prognostic m6A-related lncRNAs, m6A-related lncRNAs signature (m6A-RLRS) was constructed, and the individual riskScore of each patient with HCC was calculated according to the following equation:

$$riskScore=\sum_{i=0}^nE_i\ast C_i$$

where Ei was the expression of the selected lncRNA, Ci was the approximated regression coefficient of the corresponding lncRNA.

Next, we implemented time-dependent receiver operating characteristic (ROC) curves at 1–3 years using the “SurvivalROC” packages both in the training and testing sets to verify the performance of m6A-RLRS. Furthermore, as the riskScore of each HCC patient was calculated, patients were divided into low- and high- risk groups using the median riskScore as the threshold in the training and testing sets. The K-M survival curve was drawn to display the difference of prognosis between the two groups with the log-rank test. Moreover, stratification analyses were generated among gender (female, male), age (≤ 61 or > 61), Grade (I-II, III-IV), and TNM stage (I-II, III-IV) to confirm that the m6A-RLRS was consistent across several subgroups. Also, the riskScore of the different subgroups was compared using Wilcoxon signed-rank test to perform an internal verification.

### External validation of m6A-related lncRNA signature

External validation is decisive when constructing prognostic signatures. The expression profile data of the genes included in the m6A-RLRS were extracted from the FAHWMU set (*n* = 60) and substituted into the equations for risk score calculation. Similarly, according to the median riskScore, all patients were divided into the low- and high-risk group. Then, ROC and K-M survival curves were implemented to verify the accuracy of the signature.

### Gene set enrichment analysis

Differentially expressed genes (DEGs) among low- and high-risk groups were identified following the standards of | log2(FC)|> 1 and FDR < 0.05 using the Wilcoxon signed-rank test in the training set. We performed the gene set enrichment analysis (GSEA), including gene ontology (GO), and Kyoto encyclopedia of genes and genomes (KEGG) analyses to further understand the potential mechanisms behind the DEGs. The adjusted *P* < 0.05 was considered statistically significant.

### Independence test and establishment of a nomogram based on riskScore and clinical predictors

Age, sex, TNM stage, pathological tumor grade, and riskScore were included in the univariate and multivariate Cox regression analyses to screen the independent OS-related parameters. The time-dependent ROC curves of all factors were drawn, and AUCs of all factors were compared with the AUC of the riskScore (m6A-RLRS). Based on the “rms” package, a novel nomogram for predicting the 1-, 2-, and 3-years OS of patients with HCC was established. The Bootstrap self-sampling method was repeated 1000 times to calculate the concordance index (C-index). The decision curve analysis (DCA) curves and calibration curves were also developed to evaluate the performance of the lncRNA-clinical nomogram.

### CeRNA network construction and functional annotation

First, we identified the target miRNAs that belonged to 61 m6A-related lncRNAs in the miRcode database. Meanwhile, differentially expressed miRNAs (DEmiRNAs) were obtained based on | log2(FC)|> 1 and FDR < 0.05 using the Wilcoxon signed-rank test in 374 HCC and 50 normal tissues, and the target DEmiRNAs were identified for further study. Second, based on these DEmiRNAs, candidate target mRNAs were found in the miRTarBase, miRDB, and TargetScan databases. Similarly, differentially expressed mRNAs (DEmRNAs) were identified according to the same criteria, and the final target DEmRNAs were determined by marching DEmRNAs to the candidate target mRNAs obtained in the above three databases. Finally, a novel lncRNA-miRNA-mRNA regulatory ceRNA network was illustrated by Cytoscape [25]. Additionally, GO, and KEGG enrichment analyses were implemented to explore the underlying functions and related pathways of the final target DEmRNAs.

### qRT-PCR analysis

Sera samples were obtained from 20 HCC patients and 20 healthy controls from the FAHWMU. The use of these serum samples was approved by the ethics committee of the FAHWMU and the informed consents were received from all subjects in this study. Next, the total RNA was extracted from the sera of HCC patients as well as healthy controls using TRIzol LS reagent. The mRNA was then reverse transcribed into cDNA using ribo SCRIPTTM reverse transcription kit. The expression level of mRNA was calibrated with glyceraldehyde-3-phosphate dehydrogenase (GAPDH). SYBR Green master mix was added, and then real-time PCR was carried out using a 7500 rapid quantitative PCR system (Applied Biosystems, USA). The CT value of each well was recorded, and the relative quantification of the amplified products was performed using the 2 − ΔCt method.

### Statistical analysis

All statistical analyses were generated through R software v4.0.2, SPSS software v26.0, and GraphPad Prism 7.0. We performed the student’s t-test to compare the mean, standard error of the mean for continuous variables, and the chi-square test and fisher exact test to compare the classified variables between different groups. The P-value (two-sided) < 0.05 was used as the significant threshold.

## Results

### Prognostic value of m6A-related lncRNAs in HCC

**Fig. 1:**
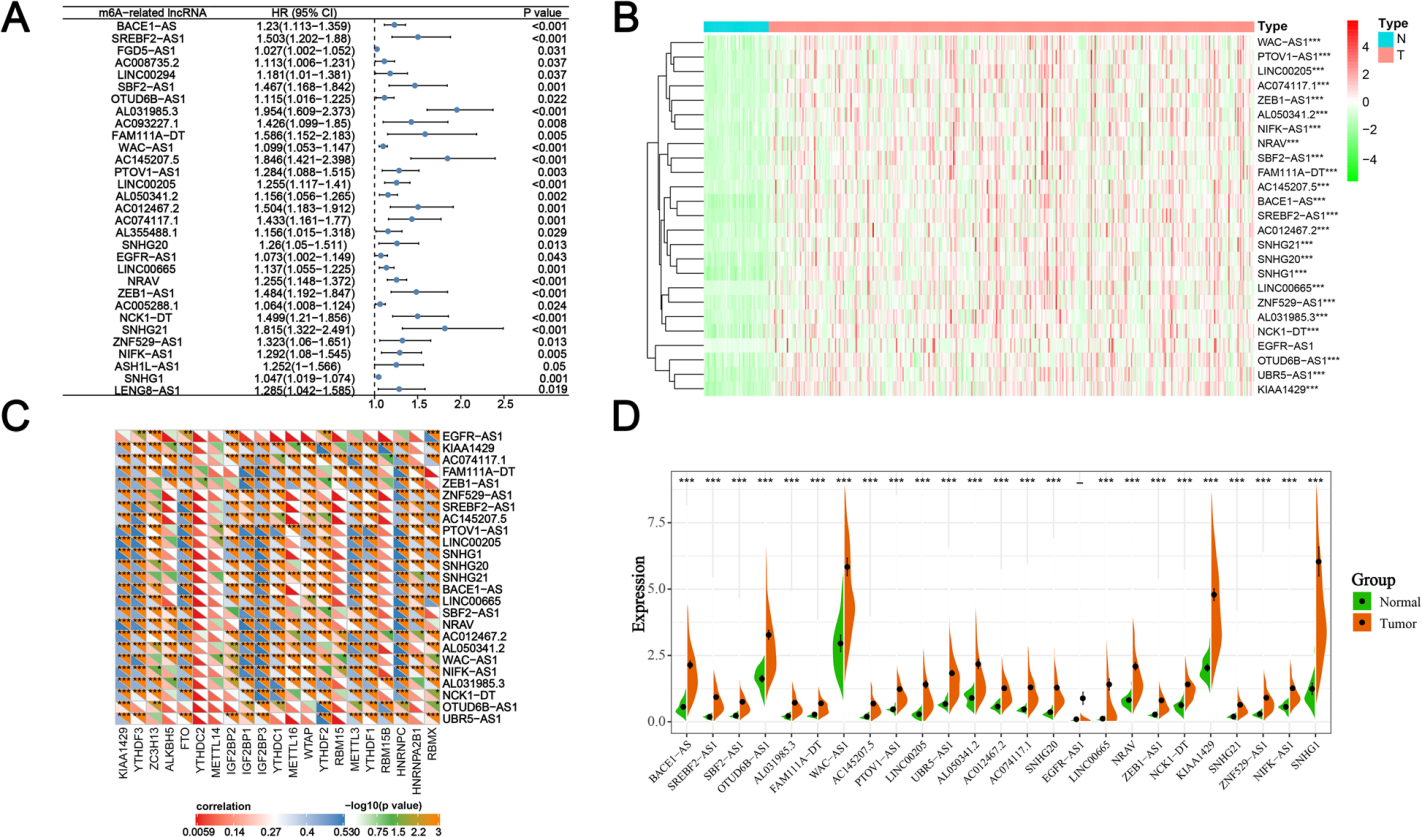
Survival, differential, and correlation analyses of m6A-related lncRNAs. **A** Forest plots for hazard ratios (HRs) of 25 prognostic m6A-related lncRNAs. **B** Heat map of 25 prognostic m6A-related lncRNAs in HCC (374) and normal tissues (50); the green to red spectrum indicates low to high gene expression. **C** Correlation matrix of interactions between 21 m6A regulators and 25 lncRNAs. **D** Violin plot of 25 prognostic m6A-related lncRNAs; green represents normal tissues, and orange represents HCC

The flow chart of our study is shown in Supplementary Fig. 1. Pearson correlation analysis was performed to mine lncRNAs related to one or more of the 21 m6A regulators, and 61 m6A-related lncRNAs were revealed (Supplementary Table 2). Then, the univariate Cox regression analysis determined 25 prognostic m6A-related lncRNAs for the following study, whose upregulation would lead to a significantly worse OS (Fig. 1A). Of the 25 prognostic m6A-related lncRNAs, 24 lncRNAs were found to be significantly upregulated in HCC, as shown in the heat map and violin plot (Fig. 1B, 1D). It was found that a lncRNA not only presented a remarkable correlation with one m6A regulator but also other regulators, which showed a comprehensive and complicated regulatory relationship between m6A modification and lncRNA (Fig. 1C). These results showed that m6A-related lncRNAs may play a significant role in the tumorigenesis and progression of HCC.

### Two m6A-related lncRNAs based HCC subtypes were significantly related to prognosis, TME characteristics, and immune features

**Fig. 2:**
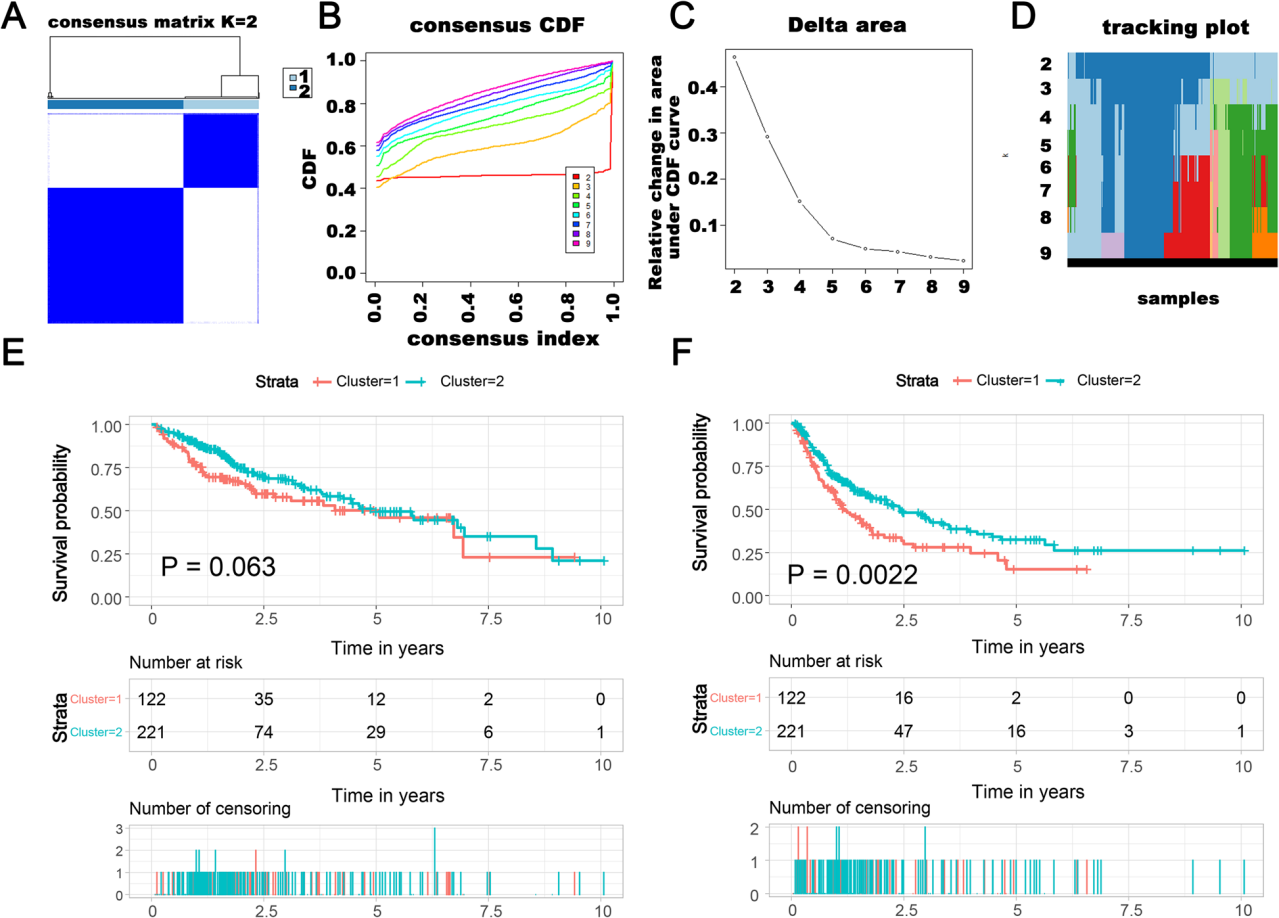
m6A-related lncRNA subtypes classification and verification. **A**-**D** Unsupervised clustering of 343 HCC cases based on 61 m6A-related lncRNAs. **E** K-M survival analysis of OS status of HCC patients in two HCC subtypes. **F** K-M survival analysis of PFS status of patients with HCC in two subtypes

The m6A-related lncRNAs profiling showed that m6A-related lncRNAs were dramatically heterogeneous in patients with HCC. We conducted an unsupervised consensus analysis of 343 HCC cases based on 61 m6A-related lncRNAs to understand the molecular heterogeneity of HCC from the perspective of m6A modification of lncRNA. The results indicated that k = 2 was more reasonable (Subtype C1: *n* = 122, 35.6%, and Subtype C2: *n* = 221, 64.4%), and all the samples were divided into two HCC subtypes, with less correlation between two subtypes (Fig. 2A-D). We compared the OS and PFS among the two subtypes of patients via the KM survival analysis to detect whether there was an association between the different subtypes and clinical outcome. Patients in the Subtype C2 had longer OS than Subtype C1, but not significantly (*P* = 0.063), and had significantly longer PFS than Subtype C1 (*P* = 0.0022) **(**Fig**. ****2**E-F).

**Fig. 3:**
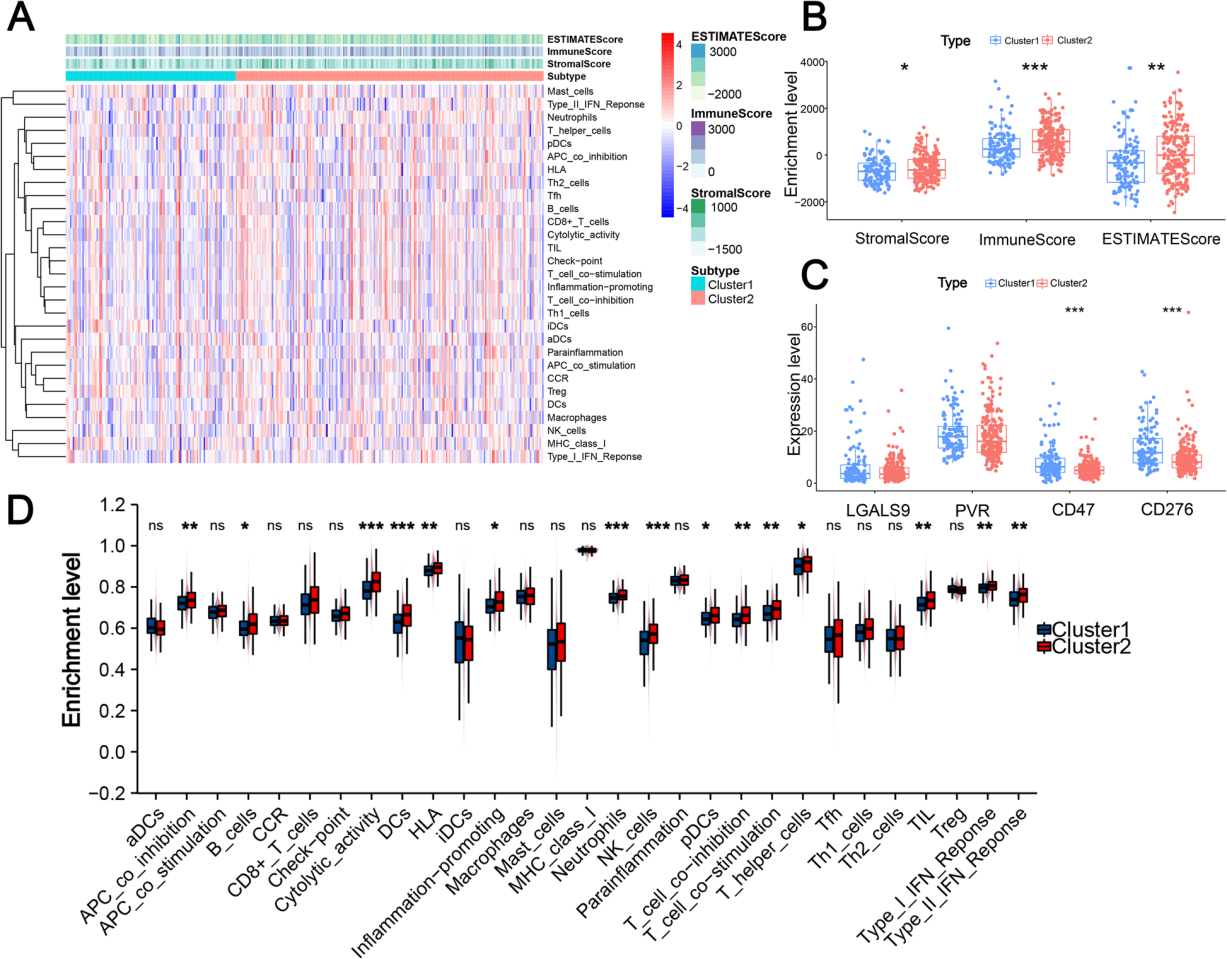
Comparison of the tumor microenvironment and immune features among the two different subtypes. **A** Heatmap of 374 cases ordered by subtypes and the relationships with TME-related scores and immune features. **B** Three types of TME-related scores between these two HCC subtypes. **C** Comparison of four immune checkpoints among the two subtypes. **D** Comparison of 29 immune-associated gene sets between these two subtypes

Moreover, we further investigated whether m6A-related lncRNA based HCC subtypes showed different immune patterns, and the Wilcoxon signed-rank test indicated that Subtype C2 with favorable prognosis had higher TME-related scores (Fig. 3B), including stromal score (*P* < 0.05), immune score (*P* < 0.001), and estimate score (*P* < 0.01). Additionally, out of four immune checkpoints, the distribution of CD47 (*P* < 0.0001) and CD276 (*P* < 0.0001) were not random (Fig. 3C), and these two immune checkpoints had higher expression in Subtype C1, which indicated that patients with HCC with subtype C1 were more suitable for targeted therapy with immune checkpoint inhibitors (ICIs) for better prognosis. Next, we compared the infiltration levels of 29 types of immune-associated gene sets between two HCC subtypes to evaluate the correlation between m6A-related lncRNA and additional immune features. The results showed that the infiltration level of all 15 significant immune-associated gene sets was higher in patients with HCC with Subtype C2, including APC-co-inhibition, B cells, DCs, Inflammation promoting, cytolytic activity, HLA, neutrophils, T cell co-inhibition, NK cells, T cell co-simulation, pDCs, T-helper-cell, TIL, Type I IFN Response, and Type II IFN Response (Fig. 3A and D). Thus, the m6A-related lncRNAs included in our study exhibited discernable patterns in prognosis, TME characteristics, and immune features of HCC patients.

### Construction and validation of the m6A-related lncRNA signature

**Fig. 4:**
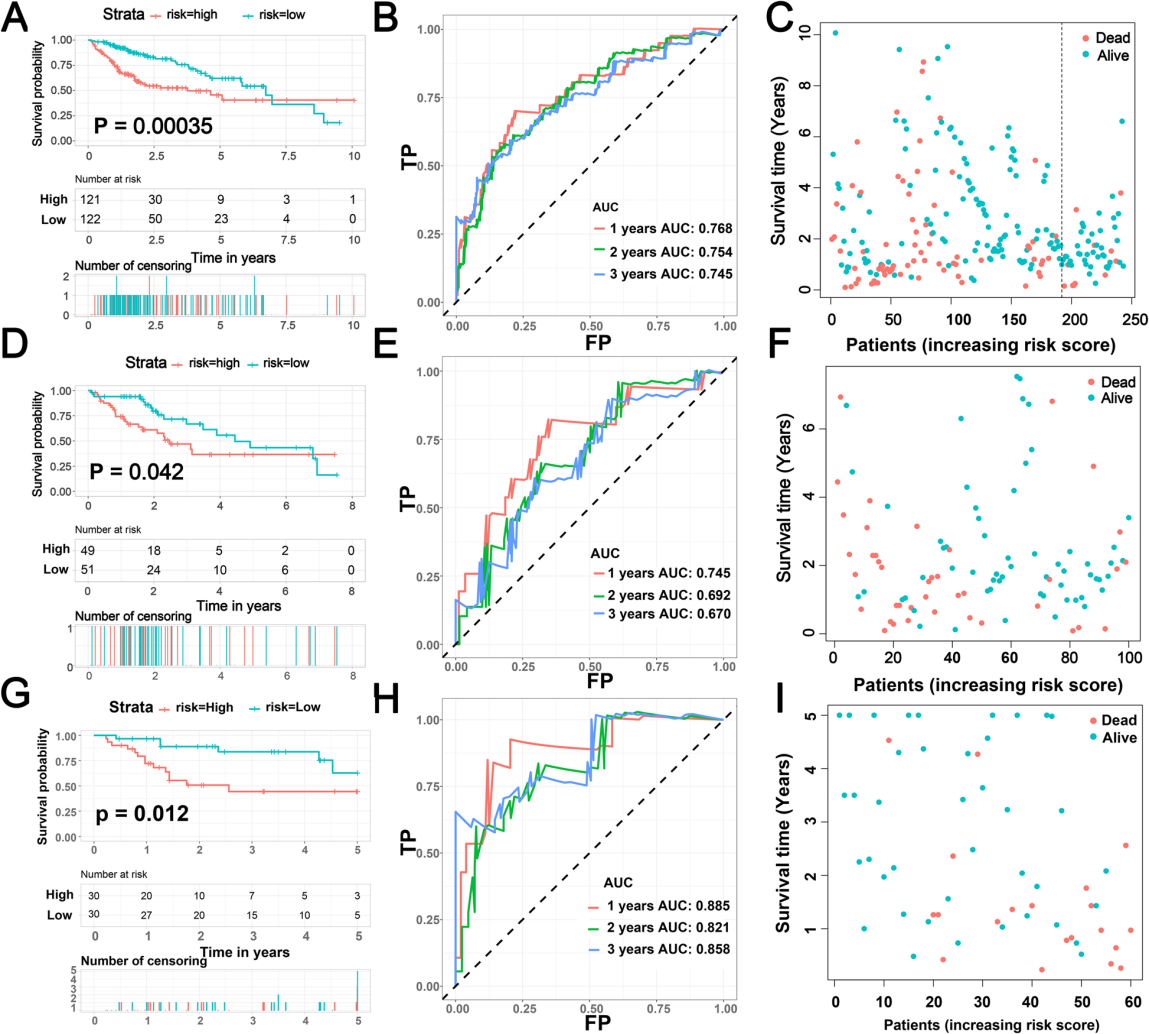
Construction and external validation of the m6A-related lncRNA signature. **A**, **D**, **G** Kaplan–Meier survival curves showing the different OS status in the training set, testing set, and FAHWMU cohort between the low-risk and high-risk groups. **B**, **E**, **H** Time-dependent ROC curves of the training set, testing set and FAHWMU cohort signatures at 1, 2, and 3 years. **C**, **F**, **I** Scatter plots of survival status for patients in the training set, testing set, and FAHWMU cohort

The identification of a robust biomarker for the early diagnosis of HCC and underlying therapeutic targets is still a key problem. Next, we generated multivariate Cox regression analysis on the basis of the 25 prognostic m6A-related lncRNAs in the training set to establish the m6A-RLRS for predicting the prognosis of HCC patients. We finally obtained four m6A-related lncRNAs and developed a prognostic m6A-RLRS (Supplementary Table 3). For each patient in the training and testing sets, a riskScore was calculated according to the coefficient and expression of each lncRNA (riskScore = 0.804* AL031985.3 + 0.521* AC145207.5–0.240* PTOV1-AS1 + 0.232*NRAV). Figure 4B and E show the ROC curves at 1-, 2-, and 3-years for the m6A-RLRS, and the corresponding AUCs were 0.768, 0.754, and 0.745 in the training set, and 0.745,0.692, and 0.670 in the testing set, implying that the m6A-RLRS could serve as a precise tool for the prognostic evaluation in patients with HCC. Figure 4C and F show the riskScore and survival status distribution. Additionally, according to the median riskScore, patients with HCC in the training and testing sets were divided into low- and high-risk groups, respectively, and the K-M curves depicted that HCC patients with higher riskScore had a worse OS than patients with lower riskScore in the training and testing sets (Fig. 4A and D). Moreover, we also verified this signature in the FAHWMU set (*n* = 60). Fig. 4I shows the riskScore and survival status distribution. Similarly, the patients with high risk had worse OS (*P* = 0.012) and the AUC values for 1–3 year were 0.885, 0.821, and 0.858 (Fig. 4G and H). The above analyses indicate the m6A-RLRS possesses a stable and robust predictive prognosis ability.

### Subgroup analysis of the m6A-related lncRNA signature

**Fig. 5:**
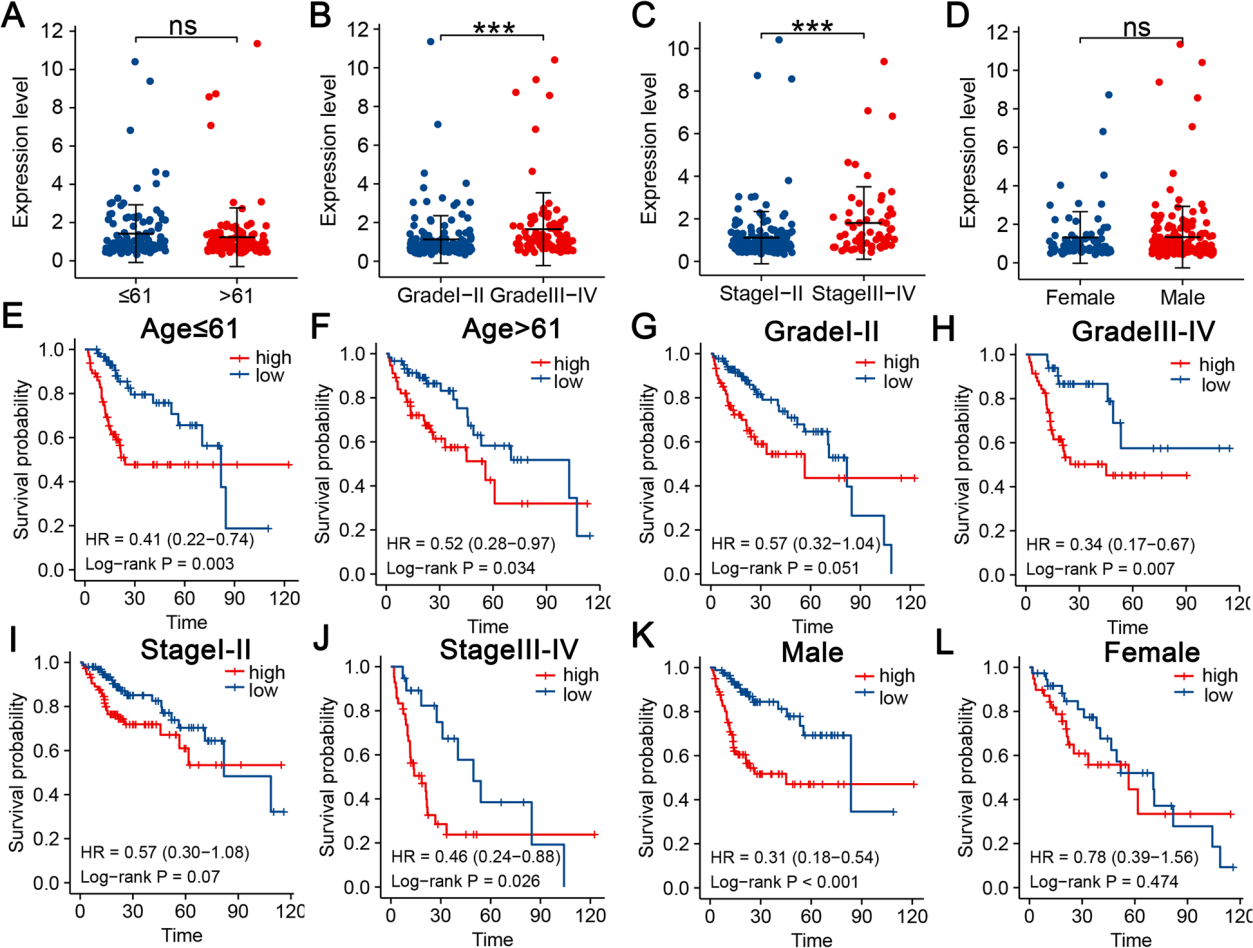
Subgroup analysis of the m6A-related lncRNA signature. **A**-**D** Comparison of the riskScore of patients with HCC with different clinicopathological features (including age, gender, tumor histology grade, and TNM stage). **E**-**L** K-M survival analysis to investigate the applicable HCC population of the m6A-RLRS

We aimed to determine whether clinical characteristics were associated with the riskScore, and the result indicated that patients with HCC with tumor histology grade III-IV and TNM stage III-IV had higher riskScores **(**Fig. 5B-C), while the riskScore were not related to age and gender (Fig. 5A and D). KM survival analyses were further implemented in subgroups based on four clinical variables to further explore the applicable HCC population of the m6A-RLRS. We confirmed that the m6A-RLRS had accurate predictive ability for HCC patients with different ages (≤ 61 or > 61) and different tumor histology grades (I-II or III-IV) **(**Fig. 5E-H). Similarly, the m6A-RLRS was suitable for patients with HCC with TNM stage III-IV but not for TNM stage I-II (*P* = 0.07) (Fig. 5I-J). Additionally, m6A-RLRS was extremely significant for male patients with HCC (*P* < 0.0001), but it had no significance for female patients (*P* = 0.47) (Fig. 5K and L). These results show that the riskScore of RLRS is associated with tumor progression and could predict the outcome of multiple subgroups of people except women.

### Gene set enrichment analysis among low- and high-risk groups

**Fig. 6:**
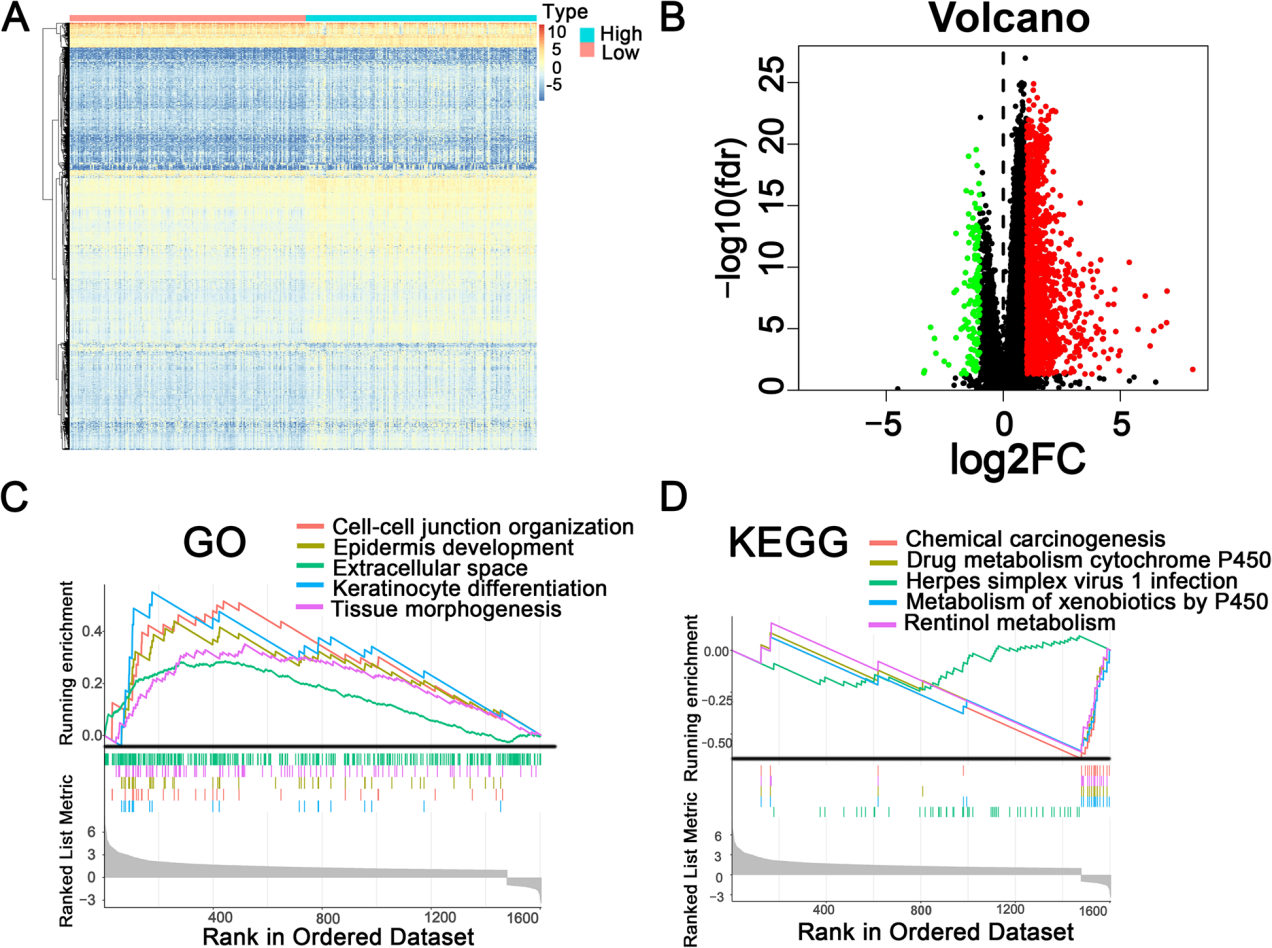
Enrichment analysis of 1611 differential expressed genes (DEGs) between low- and high-risk groups of m6A-RLRS. **A** Heatmap of 1611 DEGs in HCC and normal tissues; the blue to red spectrum exhibits low to high expression. **B** Volcano plots of 1611 DEGs; the blue point showed that the |log2FC| of the gene was > 1, while the red point indicates the opposite. **C** Top five most significant terms in GO analysis of GSEA. **D** Top five most significant terms in KEGG pathway analysis of GSEA

We screened 1611 differentially expressed genes (DEGs) to investigate potential biological mechanisms involved in molecular heterogeneity between low- and high-risk groups (Supplementary Table 4) (Fig. 6A-B). We found that most of the DEGs were enriched in cell–cell junction organization, epidermis development, extracellular space, keratinocyte differentiation, and tissue morphogenesis (GO) (Fig. 6C-D). Furthermore, KEGG pathway analysis showed that DEGs were enriched in herpes simplex virus 1 infection, metabolism of xenobiotics by cytochrome P450, drug metabolism-cytochrome P450, retinol metabolism signal pathways, and chemical carcinogenesis. These results suggest that 1611 DEGs are associated with cancer development and give us novel insights into the biological mechanisms related to m6A-related lncRNAs.

### m6A-related lncRNA signature was an independent prognostic factor for HCC patients and nomogram construction

**Fig. 7:**
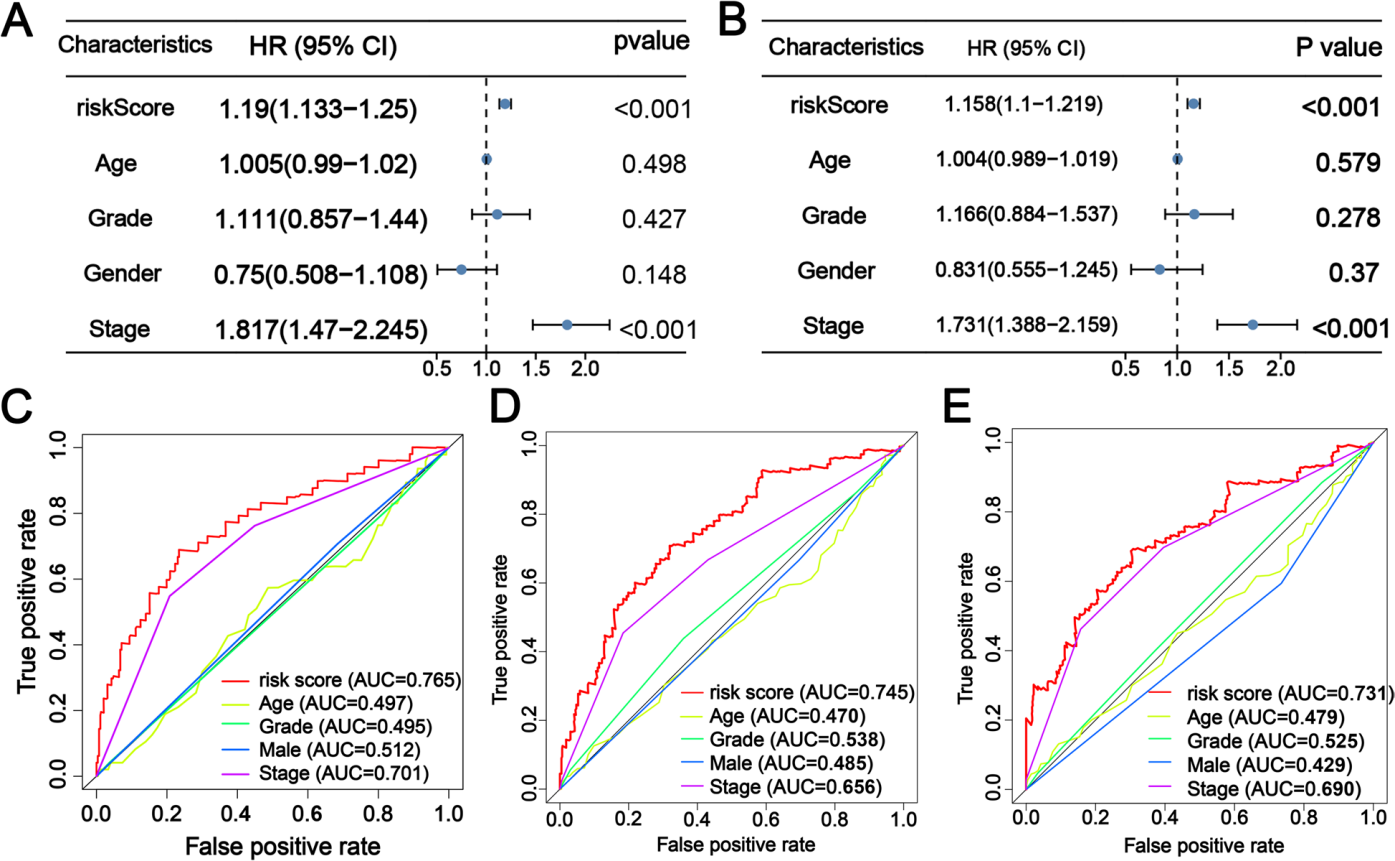
**A**, **B** Univariate and multivariate analyses showed that m6A-RLRS was an independent prognostic predictor for patients with HCC. **C**-**E** Time-dependent ROC curves for the m6A-RLRS, age, histology grade, gender, and TNM stage at 1-, 2-, 3-years

First, the univariate Cox regression analysis revelated that m6A-RLRS (HR = 1.19, 95%CI = 1.133–1.25, *P* < 0.001), and TNM stage (HR = 1.817, 95%CI = 1.47–2.245, *P* < 0.001) were OS-related variables (Fig. 7A). Additionally, the multivariate Cox analysis exhibited that m6A-RLRS (HR = 1.158, 95%CI = 1.10–1.219, *P* < 0.001) and TNM stage (HR = 1.731, 95%CI = 1.388–2.159, *P* < 0.001) were two independent OS-related variables (Fig. 7B). Subsequently, we further compared the discrimination between m6A-RLRS and other predictors, including age, histology grade, gender, and TNM stage. The results showed the AUC of signature was higher than the AUCs of all the others at 1-, 2-, and 3-years (Fig. 7C-E). These results indicate that the signature has a satisfactory ability to assess the prognosis of patients with HCC.

**Fig. 8:**
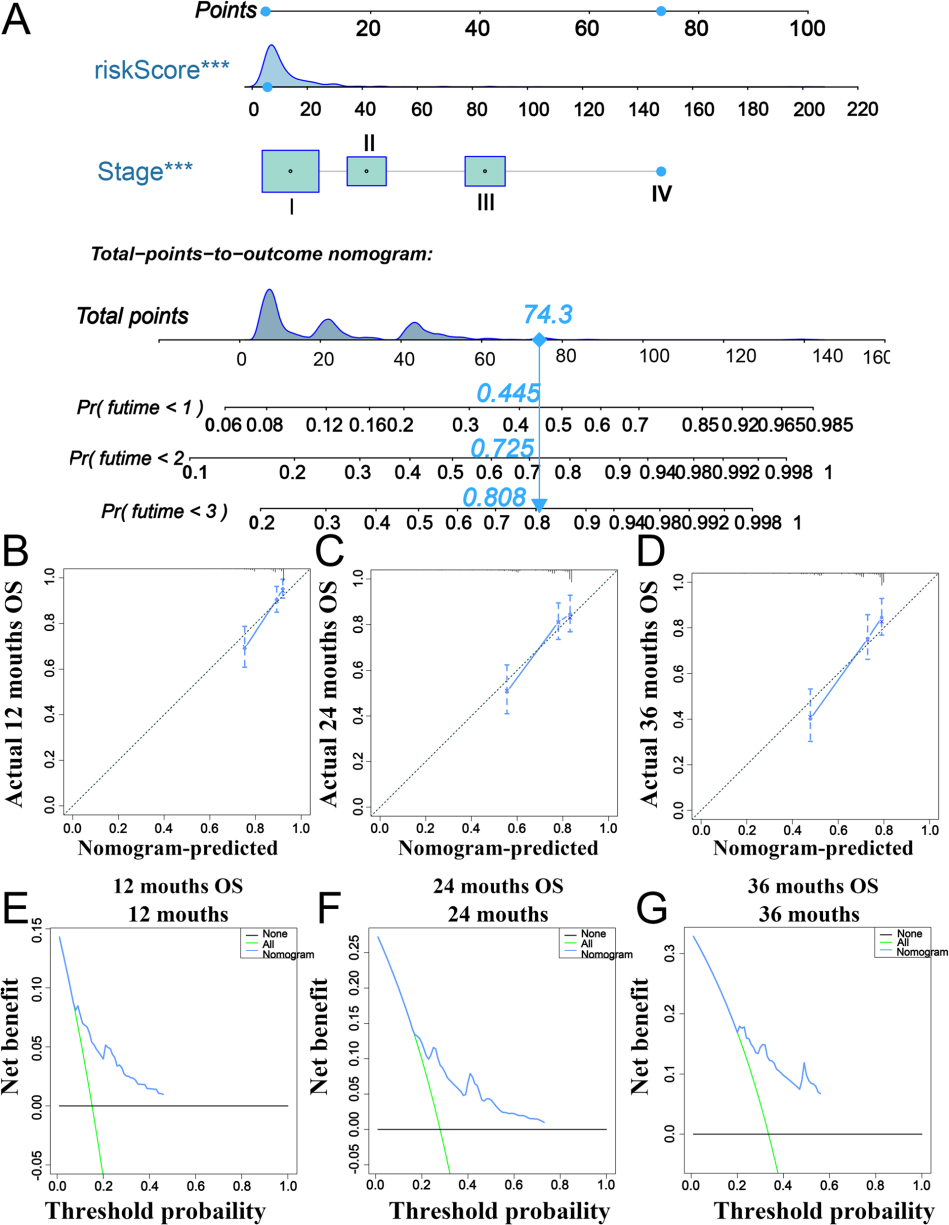
Construction and validation of a nomogram based on the m6A-RLRS and TNM stage. **A** A novel nomogram for predicting the OS of patients with HCC. **B**-**D** Calibration curves of the nomogram at 12, 24, and 36 months. **E**–**G** DCA curves of the nomogram at 12, 24, and 36 months

Meanwhile, based on two independent OS-related factors, we developed a novel nomogram to evaluate the OS of HCC patients (Fig. 8A). The C-index was 0.703 (95%CI = 0.646–0.760). The calibration curves for the probability of 1-, 2-, and 3-years OS also exhibited a great consistency between predicted OS and the actual clinical outcome (Fig. 8B-D). Moreover, the DCA curves were also generated, and the results revealed that the nomogram presented a satisfactory efficiency for OS of patients with HCC (Fig. 8E-G). These data demonstrate that the nomogram can make the personal clinical decision and surveillance more precise.

### Establishing an m6A-related lncRNA ceRNA network and functional annotation

**Fig. 9:**
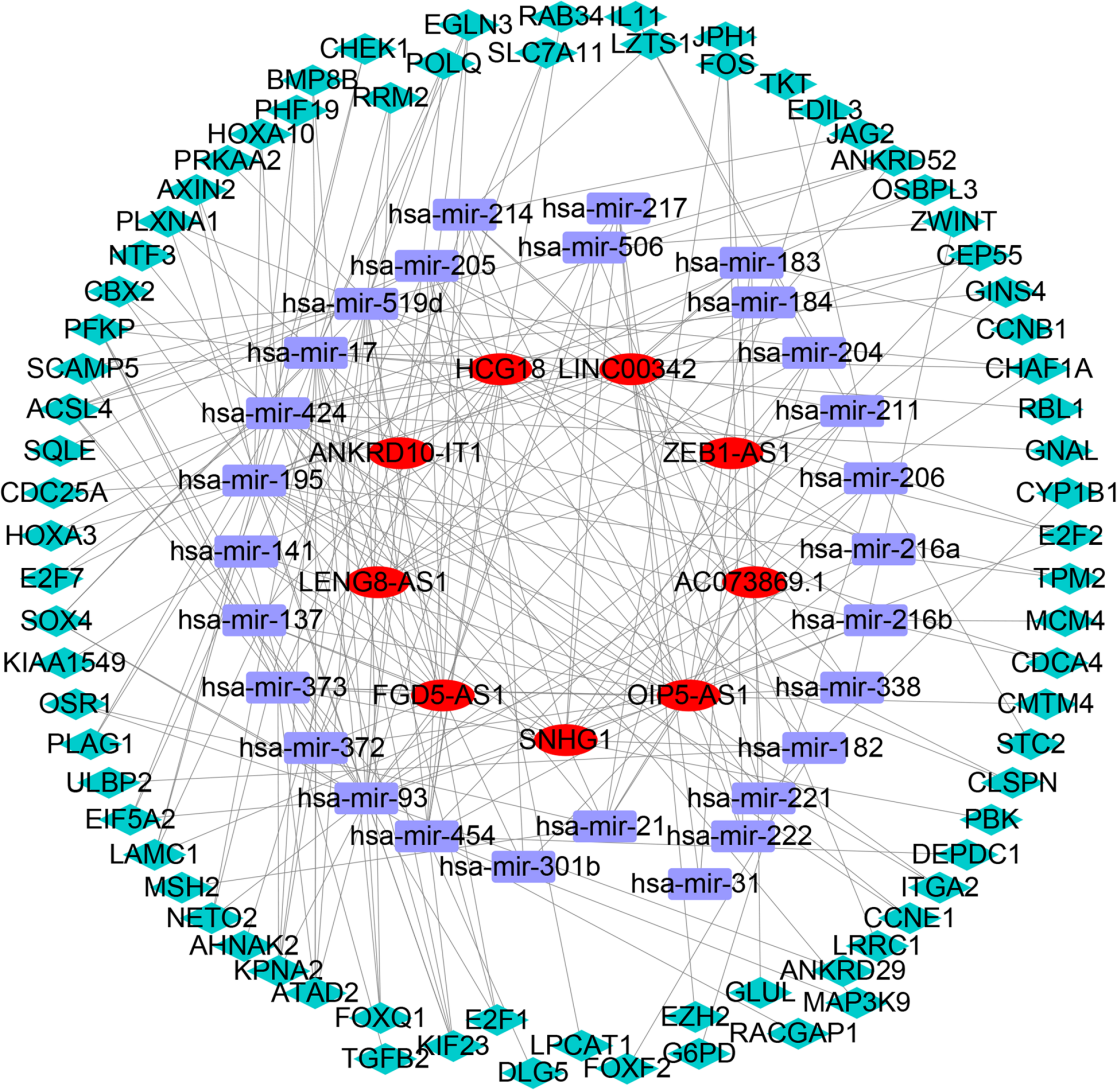
A m6A-related lncRNA ceRNA network of the nine m6A-related lncRNAs (red) and 28 target miRNAs (blue) and 75 mRNAs (green)

**Fig. 10:**
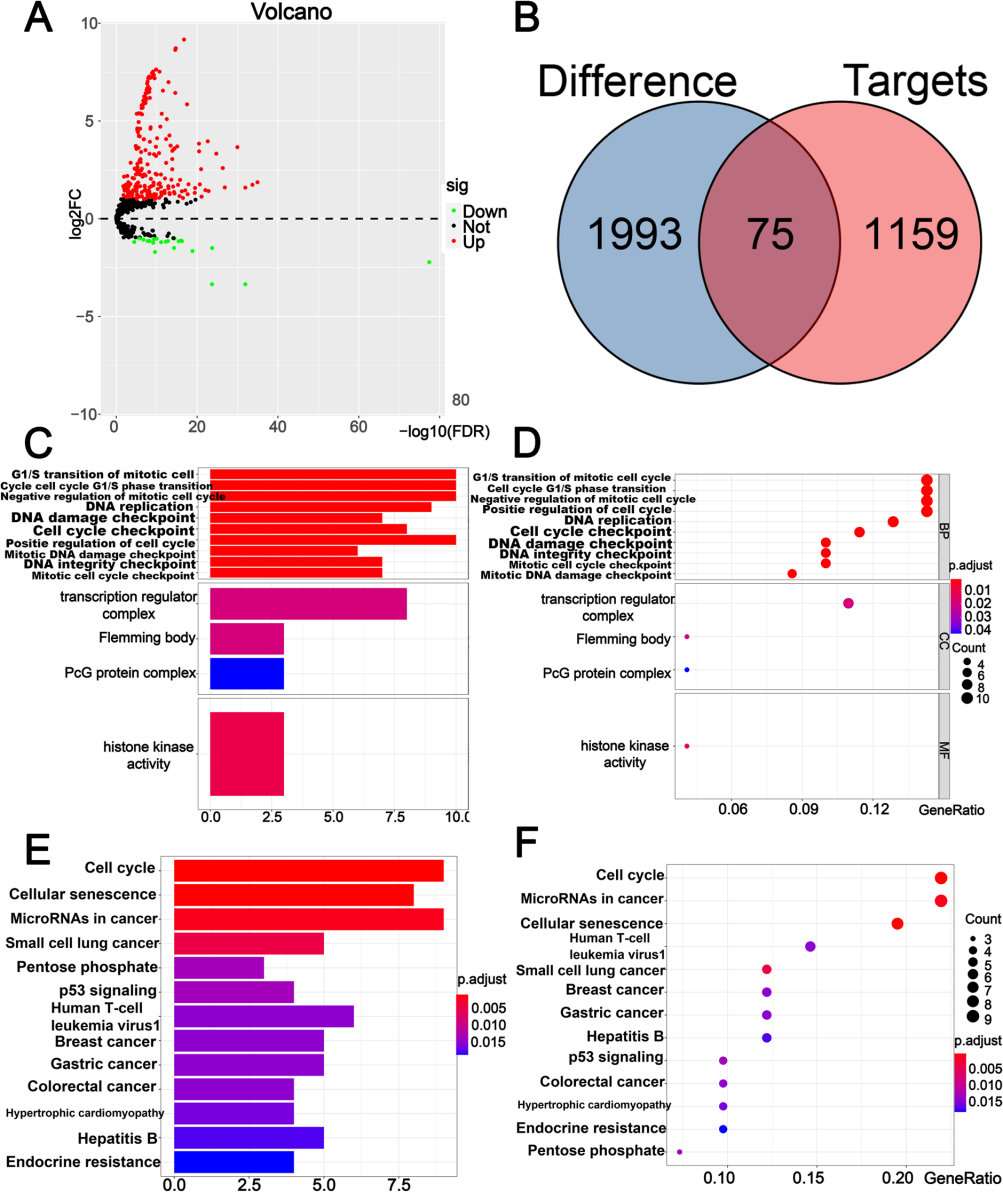
**A** Volcano plots of DEmiRNAs; the red and blue points exhibit that the |log2FC| of the gene was > 1, while the black point indicates the opposite. **B** Venn diagram indicated 75 identical mRNAs between differentially expressed genes and target mRNAs in the three databases. **C**-**D** Bar and bubble chart showed the top ten most significant terms in the GO analysis. **E**–**F** Bar and bubble chart showing the top 12 most significant terms in the KEGG pathway analysis

We constructed a ceRNA network to further investigate how the m6A-related lncRNAs regulated pivotal mRNA expression by targeting miRNAs in HCC. First, 362 pairs of interaction between 83 miRNAs and 61 lncRNAs were identified from the miRcode database (Supplementary Table 5). After determining the 251 DEmiRNAs (Supplementary Table 6) (Fig. 10A), we found 28 identical miRNAs interacting with nine key m6A-related lncRNAs and defined these miRNAs as target DEmiRNAs for further analysis. Next, we used three databases, including miRTarBase, miRDB, and TargetScan, to screen target mRNAs based on 28 DEmiRNAs, and a total of 1159 target genes was identified (Supplementary Table 7). Similarly, 1993 differentially expressed genes were identified in the 374 HCC and 50 normal liver tissues, and 75 final mRNAs were determined **(**Fig. 10B) (Supplementary Table 8). Finally, nine lncRNAs, 28 miRNAs, and 75 mRNAs were included in the m6A-related lncRNA -based ceRNA network (Fig. 9). In addition, GO functional annotation revealed that the 75 final mRNAs were mainly involved in G1/S transition of the mitotic cell cycle, cell cycle G1/S phase transition, and negative regulation of mitotic cell cycle (BP), transcription regulator complex, Flemming body, and PcG protein complex (CC), and histone kinase activity (MF) (Fig. 10C-D). KEGG pathway analysis showed that these mRNAs were mainly enriched in cell cycle, cellular senescence, and microRNAs in cancer **(**Fig. 10E-F).

### Validation of the differential expression of m6A-related lncRNA in HCC

**Fig. 11:**
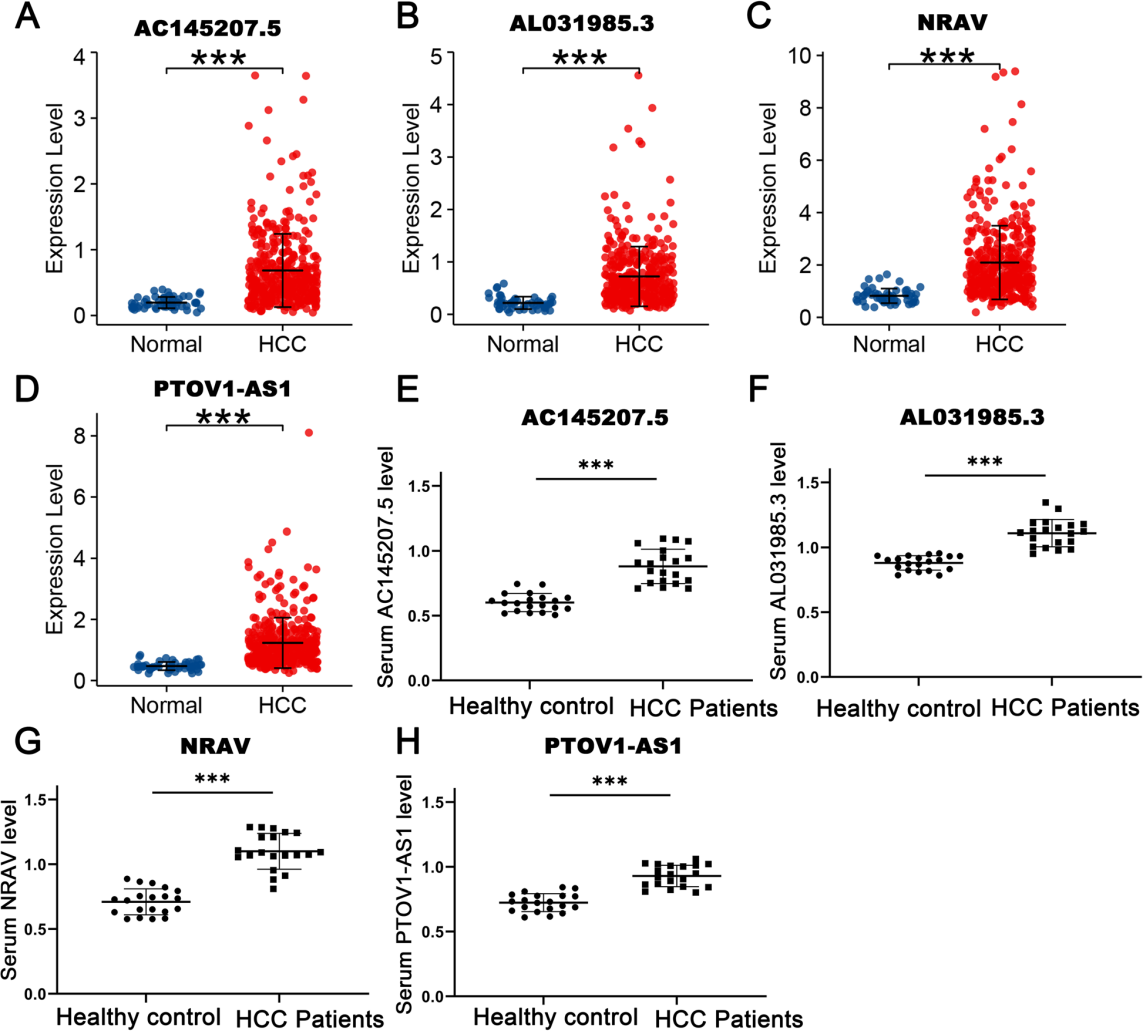
Validating the expression of four m6A-related lncRNAs in the signature by qRT-PCR. AC145207.5, AL031985.3, NRAV, and PTOV1-AS1 were all highly overexpressed in HCC, as shown in tumor tissue (**A-D**), and HCC serum (**E–H**)

qRT-PCR was performed to verify the different mRNA expression levels of four m6A-related lncRNAs in 20 HCC patients and healthy controls serum. As shown in Fig. 11, we found AC145207.5, AL031985.3, NRAV, and PTOV1-AS1 are not only highly expressed in HCC tissue as compared with adjacent tissues (Fig. 11A-D), but also in HCC serum as compared with healthy controls (Fig. 11E-H).

## Discussion

The aberrant expression of m6A regulators is known to be functionally related to cell proliferation, stress adaptation, differentiation, and resistance to therapy, all of which are biomarkers of tumorigenesis, including HCC [26–28]. Additionally, m6A modification can affect lncRNA splicing and maturation involved in the progression and initiation of cancer [21, 29]. Zheng et al. found that lncRNA FAM225A, which is positively regulated by METTL3 and acts as a ceRNA, can promote nasopharyngeal carcinoma tumorigenesis and metastasis by sponging miR-590-3p/miR1275 and upregulating ITGB3 [30]. METTL3-induced lncRNA RP11 has been shown to trigger the dissemination of cells via post-translational upregulation of Zeb1 in colorectal cancer [31]. Regarding clinical application, m6A-related lncRNAs can be regarded as the potential targets for cancer diagnosis, prognosis, and treatment. However, existing studies have mainly focused on only one or two m6A regulators and tumor cell types, while exploring novel targeted anti-tumor drugs requires the highly coordinated interaction of multiple tumor suppressor factors. However, Li et al. and Yu et al. examined the role of m6A-related lncRNA in HCC prognosis [32, 33]. Here, we identified m6A-related lncRNAs based on different correlation coefficients and comprehensively evaluated the m6A modification of lncRNA, which opened up new prospects for establishing effective prognostic models and providing novel therapeutic targets for patients with HCC. First, 61 m6A-related lncRNAs were obtained, of which 25 lncRNAs were identified to have prognostic significance. Additionally, two m6A-related lncRNA-based HCC subtypes were identified, which revealed that m6A-related lncRNAs exhibited discernable patterns in HCC and had a robust association with prognosis, TME characteristics, and immune features. Thus, we constructed an m6A-RLRS and a compressive nomogram to quantify the lncRNA m6A modification patterns of an individual patient and an original ceRNA regulatory network to illustrate potential mechanisms.

We performed a systematic analysis of m6A-related lncRNA based clustering of HCC to further understand the molecular heterogeneity of HCC from the perspective of m6A modification of lncRNA and determine whether these 61 m6A-related lncRNAs had clinical significance in HCC. Two HCC subtypes with significant survival differences were identified in our research. As the favorable prognosis cluster, Subtype C2 had a higher stromal score, immune score, and estimate score, while Subtype C1 was characterized by the suppression of immunity with lower scores. Zhang et al. indicated that stromal and immune cells enrolled in the TME could obstruct signal transduction between tumor cells, damage tumor cell metabolism, and finally inhibit tumor proliferation and invasion [34–36]. As for Subtype C1 with a worse prognosis, we speculated that a lower load of immune cells was associated with immune tolerance, escape, and quiescent T cells [37]. Consistent with the above conclusion, we also revealed that 15 of 29 immune-related gene sets had remarkably higher infiltration levels in subtype C2, which improved the effectiveness of the systemic treatment for patients with HCC [38]. Moreover, recently, immune checkpoint inhibitors (ICIs), boosting T cell activation through various mechanisms and reversing the exhausted phenotype of TME infiltrating lymphocytes, have reshaped the treatment of cancer [39]. Our study revealed that two immune checkpoints, including CD47 and CD276, had significantly higher expression levels in subtype C1, which indicated that patients in subtype C1 were more positively responsive to combination immunotherapy with targeted ICIs, to get a better OS [40, 41]. Thus, our work suggested that lncRNA m6A modification reflected the infiltration of immune cells and related biological processes, meanwhile investigating immune cell distribution in individuals provided key insights into tumor progression, immune status, and prognosis [42].

Further, of the 61 m6A-related lncRNAs, we identified 25 prognostic lncRNAs in 343 patients with HCC, four of which were included to establish the m6A-RLRS with robustness and stability. The AUCs showed that the signature performed well in predicting the OS of patients with HCC at 1, 2, and 3 years both in the training and testing sets. Our model had prospective significance in clinical utilization of HCC, and it had better predictive power than the TNM stage, the most important metric for assessing patient prognosis in current clinical practice. Moreover, the expression levels of four m6A-related lncRNAs were validated by qRT-PCR. Among the four m6A-related lncRNAs enrolled in the signature, AL031985.3 and NRAV were found to be associated with prognosis, which had important clinical implications. AL031985.3, a tumor-related lncRNAs highly expressed in lung cancer, was recently found to be involved in immune pathways and served as an accurate biomarker to assess the patients with HCC [43]. Additionally, our study revealed that AL031985.3 was positively correlated with the expression of the m6A demethylase HNRNPA2B1 and the role of their interaction in the pathogenesis of HCC development deserved further investigation. LncRNA negative regulator of antiviral response (NRAV), defined as a key regulator of the innate antiviral immunity, was first found to be dramatically downregulated during infection and modulate antiviral response by suppressing interferon-stimulated gene transcription and regulating vesicle transportation [44, 45]. NRAV promoted tumor cell growth by regulating vesicle transport and inhibiting the activation of the immune system, leading to a poor prognosis of patients with HCC [46]. Although details of the relationships between cancer and AL031985.3, AC145207.5, and PTOV1-AS1 are unclear, our study laid the foundation that m6A regulators targeted these three lncRNAs to participate in HCC tumorigenesis and progression.

Recent studies have shown that lncRNAs might function through the signaling axis of lncRNA-miRNA-mRNA, which is involved in the occurrence, development of tumors, and the formation of TME. A ceRNA network, which included 9 lncRNAs, 28 miRNAs, and 75 mRNAs, was finally obtained through the above studies to explore the role of m6A-related lncRNAs modification in HCC. Among nine lncRNAs enrolled into the network, four were found to be associated with tumorigenesis, and further experiments need to be performed to verify the role of the other genes. Zhang et al. found that lncRNA SNHG1 served as the non-degradable sponge for miR-338, which promoted the expression of proto-oncogene CST3 in primary esophageal cancer cells [47]. Jin et al. determined that lncRNA ZEB1-AS1 silencing could inhibit cell proliferation and induced the apoptosis of colorectal cancer via regulating miR-205 and YAP1 [48]. Guo et al. found that OIP5-AS1 regulated ovarian cancer progression via modulating miR-137/ZNF217 signaling [49]. Similarly, Cai et al. indicated that lncRNA FGD5-AS1 inhibited oxidative stress and apoptosis by upregulating RORA via miR-195 to inhibit hypoxia injury in human cardiomyocytes [50]. Additionally, functional annotation determined that 75 target mRNA were mainly enriched in cancer-related biological processes, including mitotic cell cycle, p53 signaling pathway, DNA damage checkpoint, and pentose phosphate pathway [51–54]. Thus, our research provided new insights into exploring potential molecular and regulatory mechanisms of lncRNA m6A.

Nevertheless, the current study had the following limitations. First, the identification of m6A-related lncRNAs was based on their expression correlation with m6A regulators, which needed further experimental verification. Second, due to the lack of available publicly lncRNA expression data in GEO and ICGC databases, the training and testing sets were derived from the same retrospective study, which had an inherent bias. Besides, the m6A-RLRS may only accurately predicted survival status at 1–3 years due to the small sample size of this study and the relatively short survival time of the patients. Additionally, although the expression levels of m6A-related lncRNAs have been verified by qRT-PCR, the functions of the m6A-related lncRNAs and their interactions with m6A regulators in HCC should be further investigated both in vitro and in vivo.

## Conclusion

Thus, we performed a systematic evaluation of the underlying regulatory mechanisms of m6A-related lncRNAs and their roles in TME constitution and tumor progression of HCC. An m6A-related lncRNA signature, a comprehensive nomogram, and an original ceRNA network were constructed. Future in-depth studies are required to explore the interactions and functions of lncRNA and m6A modifications in HCC.

## Supplementary Information

**Additional file 1: Supplementary Fig. 1**. The workflow of this study.

**Additional file 2: Supplementary Table 1**. Baseline clinical characteristics of HCC patients in the TCGA and FAHWMU cohorts.

**Additional file 3: Supplementary Table 2**. The results of correlation analysis of m6A regulators and lncRNAs.

**Additional file 4: Supplementary Table 3**. The results of Cox analyses for genes in the final signature.

**Additional file 5: Supplementary Table 4**. 1611 differentially expressed genes (DEGs) between low- and high-risk groups.

**Additional file 6: Supplementary Table 5**. 362 pairs of interaction between 83 miRNAs and 61 lncRNAs.

**Additional file 7: Supplementary Table 6**. 251 differentially expressed miRNAs between low- and high-risk groups.

**Additional file 8: Supplementary Table 7**. 1159 target genes of 28 differentially expressed miRNAs.

**Additional file 9: Supplementary Table 8**. 75 final targets mRNAs in the CeRNA network.

## Data Availability

Publicly available database analyzed in this study can be found in the The Cancer Gernome Altas (https://portal.gdc.cancer.gov/). The dataset of external cohort from our hospital is available from the corresponding author upon reasonable request.

## Abbreviations

M6A: N6 methylandenosine
TME: Tumor microenvironment
HCC: Hepatocellular carcinoma
m6A-RLRS: M6A related lncRNA signature
OS: Overall survival
PFS: Progression-free survival
ncRNA: Noncoding RNAs
lncRNA: Long noncoding RNA
XIST: X-inactive specific transcript
ceRNA: Competing endogenous
RNA-seq: RNA-sequencing
KM: Kaplan–Meier
ssGSEA: Single-sample gene set enrichment analysis
ROC: Receiver operating characteristic
DEGs: Differentially expressed genes
GO: Gene ontology
KEGG: Kyoto encyclopedia of genes and genomes analyses
C-index: Concordance index
DCA: Decision curve analysis
DEmiRNAs: Differentially expressed miRNAs
DEmRNAs: Differentially expressed mRNAs
ICIs: Immune checkpoint inhibitors
