# Supplementary figures and images for: M6A-related lncRNAs predict clinical outcome and regulate the tumor immune microenvironment in hepatocellular carcinoma
Source: BMC Cancer. 2022 Aug 9;22:867. doi: 10.1186/s12885-022-09925-2 (PMC9361634; doi:10.1186/s12885-022-09925-2)

**Figure legend**


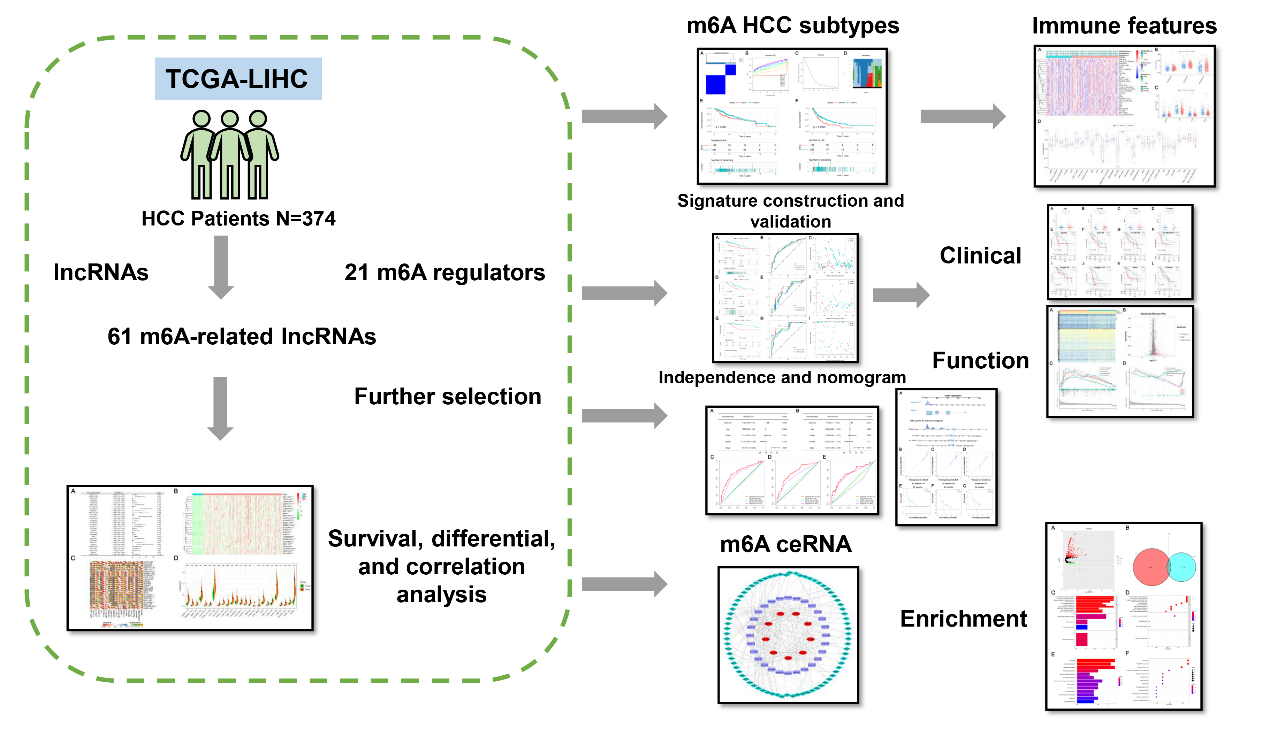


**Supplementary Fig. 1:** The workflow of this study.

Supplement: Supplementary file 1 — Additional file 1: Supplementary Fig. 1. The workflow of this study. [file 12885_2022_9925_MOESM1_ESM.docx]
