# Supplementary material for: M6A-related lncRNAs predict clinical outcome and regulate the tumor immune microenvironment in hepatocellular carcinoma
Source: BMC Cancer. 2022 Aug 9;22:867. doi: 10.1186/s12885-022-09925-2 (PMC9361634; doi:10.1186/s12885-022-09925-2)
Supplement: Supplementary file 2 — Additional file 2: Supplementary Table 1. Baseline clinical characteristics of HCC patients in the TCGA and FAHWMU cohorts. [file 12885_2022_9925_MOESM2_ESM.docx]

| Supplementary Table 1. Baseline clinical characteristics of HCC patients in the TCGA and FAHWMU cohorts | | | | | | |
| --- | --- | --- | --- | --- | --- | --- |
|  | Training group | FAHWMU | P | Validation group | FAHWMU | P |
| Age, years | 59.4±13.0 | 57.8±12.5 | 0.406 | 59.4±13.59 | 57.8±12.5 | 0.481 |
| Sex |  |  | 0.658 |  |  | 0.538 |
| Male | 167 | 43 |  | 67 | 43 |  |
| Female | 76 | 17 |  | 33 | 17 |  |
| Tumor Grade |  |  | 0.441 |  |  | 0.681 |
| I | 32 | 11 |  | 21 | 11 |  |
| II | 117 | 33 |  | 44 | 33 |  |
| III | 83 | 14 |  | 29 | 14 |  |
| IV | 8 | 2 |  | 4 | 2 |  |
| Unknow | 3 | 0 |  | 2 | 0 |  |
| T stage |  |  | 0.625 |  |  | 0.523 |
| I | 121 | 37 |  | 48 | 37 |  |
| II | 58 | 12 |  | 26 | 12 |  |
| III | 53 | 10 |  | 21 | 10 |  |
| IV | 9 | 1 |  | 4 | 1 |  |
| Unknow | 2 | 0 |  | 1 | 0 |  |
| N stage |  |  | 0.470 |  |  | 0.204 |
| N0 | 172 | 47 |  | 68 | 47 |  |
| N1 | 3 | 0 |  | 0 | 0 |  |
| Unknow | 68 | 13 |  | 32 | 13 |  |
| M stage |  |  | 0.679 |  |  | 0.083 |
| M0 | 180 | 48 |  | 65 | 48 |  |
| M1 | 2 | 0 |  | 1 | 0 |  |
| Unknow | 61 | 12 |  | 34 | 12 |  |
| TNM stage |  |  | 0.284 |  |  | 0.693 |
| I | 117 | 34 |  | 45 | 34 |  |
| II | 56 | 11 |  | 21 | 11 |  |
| III | 59 | 10 |  | 21 | 10 |  |
| IV | 2 | 0 |  | 1 | 0 |  |
| Unknow | 9 | 5 |  | 12 | 5 |  |
