# Supplementary material for: M6A-related lncRNAs predict clinical outcome and regulate the tumor immune microenvironment in hepatocellular carcinoma
Source: BMC Cancer. 2022 Aug 9;22:867. doi: 10.1186/s12885-022-09925-2 (PMC9361634; doi:10.1186/s12885-022-09925-2)
Supplement: Supplementary file 4 — Additional file 4: Supplementary Table 3. The results of Cox analyses for genes in the final signature. [file 12885_2022_9925_MOESM4_ESM.docx]

| Supplementary Table 3. The results of Cox analyses for genes in the final signature | | | | | | | |
| --- | --- | --- | --- | --- | --- | --- | --- |
| Gene | Univariate analysis | | |  | Multivariate analysis | | |
|  | HR | 95%CI | P |  | HR | 95%CI | P |
| AL031985.3 | 2.556 | 1.878-3.479 | <0.001 |  | 2.235 | 1.518-3.290 | <0.000 |
| AC145207.5 | 2.032 | 1.531-2.696 | <0.001 |  | 1.684 | 1.216-2.332 | 0.002 |
| PTOV1-AS1` | 1.269 | 1.046-1.539 | 0.016 |  | 0.787 | 0.565-1.096 | 0.156 |
| NRAV | 1.281 | 1.138-1.442 | <0.001 |  | 1.261 | 1.068-1.488 | 0.006 |
